# Comparison of adipose tissue- and bone marrow- derived mesenchymal stem cells for alleviating doxorubicin-induced cardiac dysfunction in diabetic rats

**DOI:** 10.1186/s13287-015-0142-x

**Published:** 2015-08-22

**Authors:** Hania Ibrahim Ammar, Glen Lester Sequiera, Mira B. Nashed, Rasha I. Ammar, Hala M. Gabr, Hany E. Elsayed, Niketa Sareen, Ejlal Abu-El Rub, Maha B. Zickri, Sanjiv Dhingra

**Affiliations:** Department of Physiology, Faculty of Medicine, Cairo University, Cairo, Egypt; Regenerative Medicine Program, Institute of Cardiovascular Sciences, St. Boniface Hospital Research Centre, University of Manitoba, Winnipeg, R2H2A6 Canada

## Abstract

**Introduction:**

Doxorubicin (DOX) is a well-known anticancer drug. However its clinical use has been limited due to cardiotoxic effects. One of the major concerns with DOX therapy is its toxicity in patients who are frail, particularly diabetics. Several studies suggest that mesenchymal stem cells (MSCs) have the potential to restore cardiac function after DOX-induced injury. However, limited data are available on the effects of MSC therapy on DOX-induced cardiac dysfunction in diabetics. Our objective was to test the efficacy of bone marrow-derived (BM-MSCs) and adipose-derived MSCs (AT-MSCs) from age-matched humans in a non-immune compromised rat model.

**Methods:**

Diabetes mellitus was induced in rats by streptozotocin injection (STZ, 65 mg/kg b.w, i.p.). Diabetic rats were treated with DOX (doxorubicin hydrochloride, 2.5 mg/kg b.w, i.p) 3 times/wk for 2 weeks (DOX group); or with DOX+ GFP labelled BM-MSCs (2x106cells, i.v.) or with DOX + GFP labelled AT-MSCs (2x106cells, i.v.). Echocardiography and Langendorff perfusion analyses were carried out to determine the heart function. Immunostaining and western blot analysis of the heart tissue was carried out for CD31 and to assess inflammation and fibrosis. Statistical analysis was carried out using SPSS and data are expressed as mean ± SD.

**Results:**

Glucose levels in the STZ treated groups were significantly greater than control group. After 4 weeks of intravenous injection, the presence of injected MSCs in the heart was confirmed through fluorescent microscopy and real time PCR for ALU transcripts. Both BM-MSCs and AT-MSCs injection prevented DOX-induced deterioration of %FS, LVDP, dp/dt max and rate pressure product. Staining for CD31 showed a significant increase in the number of capillaries in BM-MSCs and AT-MSCs treated animals in comparison to DOX treated group. Assessment of the inflammation and fibrosis revealed a marked reduction in the DOX-induced increase in immune cell infiltration, collagen deposition and αSMA in the BM-MSCs and AT-MSCs groups.

**Conclusions:**

In conclusion BM-MSCs and AT-MSCs were equally effective in mitigating DOX-induced cardiac damage by promoting angiogenesis, decreasing the infiltration of immune cells and collagen deposition.

**Electronic supplementary material:**

The online version of this article (doi:10.1186/s13287-015-0142-x) contains supplementary material, which is available to authorized users.

## Introduction

With a prevalence in over 382 million people, diabetes mellitus, which is presently among the top 10 killers worldwide, is projected to affect 592 million by 2035 [1]. Epidemiological evidences have shown established connections between diabetes mellitus and cancer. It is reported that in diabetic patients not only the risk of cancer is increased, but the rate of patient survival has also been found to be low [2]. Some of the probable mechanisms that have been proposed to play a role in this increased prevalence are hyperinsulinemia, hyperglycemia and chronic inflammation [3]. Doxorubicin (DOX), an anticancer drug, is regularly a part of combination therapy and acts by intercalating DNA and inhibiting the process of replication [4]. Its clinical application is limited though due to its cardiotoxic effects in normal individuals. Also it has been reported that diabetes mellitus increases accumulation of DOX in the heart and the resultant cardiac injury is far greater than in non-diabetic individuals [5]. As diabetes mellitus itself can lead to heart failure [6], using DOX in comorbid patients to treat cancer puts them at potentially increased risk of cardiac injury.

Stem cells provide a vast avenue to explore cell therapy for cardiac regeneration. Though there are a lot of candidates, mesenchymal stem cells (MSCs) have emerged as the prime ones. Several studies have demonstrated that MSCs are safe and effective for cardiac repair [7]. They retain their immune privilege when injected into myocardium and are allogenically compatible [8]. The rescue of cardiac function has been accredited to a multitude of factors, mainly their ability to secrete a wide array of paracrine factors [7], recruitment of endogenous cardiac stem cells [9], by promoting angiogenesis and by mitigating inflammation and fibrosis [7, 10]. Cardiac function has been established to be highly benefitted by vascularization, therefore increased angiogenesis in the ischemic heart is considered to be an integral part of cardiac repair [11]. Several studies have reported that MSCs secrete several pro-angiogenic and immunosuppressive factors such as placental-derived growth factor (PIGF), vascular endothelial growth factor (VEGF), fibroblast growth factor-2 (FGF-2), angiopoeitin-1, platelet-derived growth factor (PDGF), monocyte chemotactic protein-1 (MCP-1), plasminogen activator and matrix metalloproteinase-9 (MMP-9), prostaglandin E2 (PGE2) and interleukin 10 (IL-10) [12, 13].

To date, there has also been some research into mitigating DOX-induced cardiomyopathy through the application of MSCs [14, 15]. However, a more pertinent study employing relevant diabetic models is not available. This study aims to examine the capacity of MSCs to restore heart function in diabetic rats with cardiac injury following DOX administration. MSCs derived from bone marrow (BM) and from adipose tissue (AT) are currently touted to be chief prospective sources for therapeutic applications [16]. BM-MSCs are hard to obtain, given their source [17], while AT-MSCs are relatively easier and more straight forward to establish [18]. Furthermore, both BM-MSCs and AT-MSCs express the same surface markers [19]. There is broad similarity in differentiation ability between BM-MSCs and AT-MSCs [20]. AT-MSCs have displayed higher proliferation rates. The secretion of various angiogenic factors were also reported to be slightly in the two sources of MSCs. In this regard high levels of VEGF, IGF and SCF are reported to be produced in BM-MSCs, while on the other hand, AT-MSCs produce a significant amount of basic fibroblast growth factor (bFGF) [21]. Given these considerations and their present clinical applicability, both were used in this study to allow for comparison and a comprehensive approach.

## Materials and methods

This study was approved by the ethical committee of the Faculty of Medicine, Cairo University, Egypt.

### Isolation and expansion of BM-MSCs

Human bone marrow samples were obtained from healthy age-matched (25 − 40 years) adult volunteer donors after written consent and approval were obtained. All procedures were performed in Kasr Al Ainy University hospital in accordance with the code of conduct approved by the Ethics Committee of the Faculty of Medicine, Cairo University. A 5-mL bone marrow sample was aspirated from the posterior superior iliac spine. After 1:1 dilution with Hank’s balanced salt solution (Lonza, Basel, Switzerland), the bone marrow samples were layered over Ficol hypaque (Invitrogen, Waltham, Massachusetts, USA) for density gradient centrifugation. Mononuclear cells thus separated were counted and plated at a density of 500,000 cells/flask in complete medium. Cells were suspended in complete alpha minimum essential media (α-MEM; Gibco®, Burlington, USA) supplemented with 10 % fetal bovine serum (FBS; Hyclone, Logan, Utah, USA), 1 × nonessential amino acids (NEAA; Sigma-Aldrich, St. Louis, USA), 4 mM L-glutamine (Sigma-Aldrich, St. Louis, USA), and 100 U/mL penicillin, 0.01 mg/mL streptomycin sulfate (Sigma-Aldrich, St. Louis, USA) in T25 tissue culture flasks. Flasks were incubated in a humidified 5 % CO2 incubator at 37 °C. After 24 h the culture media were changed to remove non-adherent cells. For all of the experiments, only cells from the early passages (P) of culture were used (P2 to P4).

### Isolation and expansion of AT-MSCs

Human subcutaneous adipose tissues were obtained from adult age-matched healthy subjects (28 − 40 years) undergoing liposuction after written consent and approval were obtained. All procedures were performed in Kasr Al Ainy University hospital in accordance with the code of conduct approved by the Ethics Committee of Faculty of Medicine, Cairo University. Cell isolation was performed as previously published [22]. Briefly, adipose tissue biopsy samples were collected under sterile conditions in serum-free DMEM/F12 medium (supplemented with 200 μg/mL streptomycin and 200U/mL penicillin; Gibco®, Burlington, USA). Tissue samples were washed in PBS, minced, and digested with 1 mg/mL collagenase type I in 0.1 % BSA for 1 h at 37 °C. Minced samples were centrifuged at 650 *g* for 10 minutes. The pellet was treated with red blood cell lysis buffer (155 mM NH4Cl, 10 mM KHCO3, and 0.1 mM EDTA) for 10 minutes at room temperature (RT). After centrifugation (650 g for 10 minutes), the cellular pellet was filtered through a 100-μm mesh filter to remove debris. The filtrate was centrifuged, and the obtained stromal vascular fraction (SVF) was plated onto T25 cell culture flasks in complete culture medium (DMEM containing 20 % FBS, 100 μg/mL streptomycin, 100 U/mL penicillin, 2 mM l-glutamine, and 1 μg/mL amphotericin-B; Gibco®, Burlington, USA). Cells were then cultured as for BM-MSCs. For all of the experiments, only cells at early passages of culture were used (P2 to P4). In accordance with the Second Annual Meeting of the International Fat Applied Technology Society (Pittsburgh, PA, USA, 2004), the obtained plastic adherent cell stromal populations expanded from collagenase digests of adipose tissue have been termed adipose stem cells (ASCs).

### Characterization of mesenchymal stem cells

The selection of immunophenotyping was based on the International Society for Cellular Therapy (ISCT) proposal [23]. Briefly, the non-adherent hematopoietic cells were washed off, and the adherent MSCs were characterized by fluorescence-activated cell sorting (FACS; Beckman Coulter, NE15106, USA) with antibodies against CD45, CD34, CD90.1, CD44, and CD105.

### In vivo studies

#### Animals

Fifty male Wistar rats weighing 200 − 220 g were used in this study. Animals were housed at the Animal Care facility of the Faculty of Medicine, Cairo University, in chip-bedded cages at RT under a 12:12-h light–dark cycle and were given free access to standard rat chow and water for the entire duration of the study. The experimental protocol and procedures were approved by the Institutional Animal Care and Ethical Committee, Kasr Al-Ainy Faculty of Medicine, Cairo University.

#### Experimental design

Weight-matched rats (n = 10/group) were allocated into the following groups: group 1, control (C), received 0.2 mL of saline (vehicle; intraperitoneal (i.p.)), in six equal doses over 2 weeks; group 2, diabetic group (STZ), injected with streptozotocin (STZ, MP Biochemicals, CA, USA; 65 mg/kg body wt, i.p.) to induce diabetes mellitus. STZ was dissolved in sodium citrate buffer (pH 4.5) and stored at 4 °C [24]; group 3, diabetic + DOX group (STZ + DOX), received a single dose of STZ (65 mg/kg body wt, i.p.), followed after 4 weeks by adriamycin (doxorubicin hydrochloride, Pharmacia Italia, Nerviano, Italy; 2.5 mg/kg body wt, i.p.) in saline. DOX was injected in six equal doses over the period of 2 weeks to induce heart failure; group 4, bone marrow group (BM-MSCs), received BM MSCs (2 × 10^6^/mL stem cells intravenously (i.v.) into the tail vein) 4 weeks after the first DOX injection (8 weeks after the STZ injection); group 5, adipose tissue group (AT-MSCs), received AT-MSCs (2 × 10^6^/mL stem cells i.v. into the tail vein) 4 weeks after the first DOX injection (8 weeks after the STZ injection). For the detailed treatment plan, please see Figure S3 in Additional file 1.

Noninvasive blood pressure measurements and echocardiography were performed for all rats at baseline, 4 weeks after STZ injection, 4 weeks after the first DOX injection (8 weeks after STZ injection), and 4 weeks after stem cell injection (12 weeks after STZ injection). At the end of the study (12 weeks after STZ injection), blood samples were collected for serum insulin and fibrinogen measurements. Hearts were excised from rats in all treatment groups (after 12 weeks of STZ injection) for ex vivo heart perfusion as described below. Heart tissue samples were then collected for subsequent histopathological analysis and detection of injected stem cells.

#### Verification of diabetes mellitus

Blood samples were collected from rat tail veins for all studied groups at baseline and at 4, 8 and 12 weeks from STZ injection. Blood glucose was assayed by a kit supplied by Diamond Diagnostics (MA, USA).

#### Care of diabetic animals

After STZ administration, rats were closely observed during the first 48 h for hypoglycemia and release of stored insulin (data not shown). Diabetic rats were also provided with plenty of fluid to compensate for the high urine volume. Two to three STZ diabetic rats were housed per cage (21 cm high by 25.5 cm wide by 47 cm long) and housing conditions were closely monitored.

#### Arterial blood pressure measurements

The mean arterial blood pressure (ABP) was recorded in conscious rats using the tail-cuff method (Harvard 50–9331 Rectilinear recording System; Harvard Apparatus, Kent, UK). Rats were acclimated for restraint and tail-cuff inflation for 5 to 7 days before the procedure. A tail-cuff occluded with an optical pulse sensor was placed proximally on the tail. To obtain an accurate blood pressure reading, rats were allowed 5 minutes calm in the restrainers. On inflation, the cuff occluded blood flow through the tail, and on deflation the return of blood is detected by the optical pick up system and converted to an analog signal through a built-in pressure transducer. The pressure in the cuff is increased above the systolic pressure till no pulse is recorded, and then the pressure is allowed to slowly drop below the systolic, while the pulse amplitude rapidly increases. Systolic and diastolic pressure readings are then captured and displayed on a PC computer (Intracell, UK.) At least three consecutive readings were obtained and averaged for each rat.

#### Echocardiography

Echocardiography was performed to evaluate cardiac function to all five groups. The rats were lightly anesthetized with an injection of ketamine hydrochloride (25 mg/kg, i.p.) and xylazine (5 mg/kg, i.p.). An echocardiography system equipped with a 12-MHz phased-array transducer (SONOS 5500; Philips Medical System, Best, Netherlands) was placed over the left parasternal area and rocked through the heart from the apex to the base. A two-dimensional short-axis view of the left ventricle and M-mode tracings were recorded to measure left ventricular end-diastolic dimension (LVEDD) and left ventricular end systolic dimension (LVSD). Percent fractional shortening (FS) was calculated from the composite LV internal diastolic (LVEDD) and LV internal systolic (LVSD) dimensions as follows:

$$ \mathrm{F}\mathrm{S}=\frac{\mathrm{End}\hbox{-} \mathrm{diastolic}\kern0.5em \mathrm{dimension}-\mathrm{End}\hbox{-} \mathrm{systolic}\kern0.5em \mathrm{dimension}}{\mathrm{End}\hbox{-} \mathrm{diastolic}\kern0.5em \mathrm{dimension}}\kern0.5em \times 100 $$

#### Isolated heart perfusion

At the end of in vivo experiments (echocardiography), the animals were anesthetized using ketamine hydrochloride (25 mg/kg, i.p.) and heparinized by i.p. injection (1,000 IU). A left thoracotomy was performed and hearts were rapidly exposed, excised and immediately placed in ice cold Kreb-Henseleit (KH) heparinized solution. The ascending aorta was then cannulated and placed along the perfusion line of non-recirculating constant-flow Langendorff apparatus (Radnotti, Harvard apparatus, USA). The apparatus was maintained at 37 °C. The duration between excision and perfusion of the hearts did not exceed one minute. Hearts were then perfused using KH buffer with the following concentration (in mM): 25 NaHCO_3_, 4.7 KCl, 118.5 NaCl, 1.2 MgSO_4_, 1.2 KH_2_PO_4_, 2.5 CaCl_2_ and 10 glucose, pH 7.4 (Sigma Aldrich, MO, USA). Perfusion was maintained at a constant flow of 16 mL/min at 37 °C and aerated with a gas mixture (95 % O_2_, 5 % CO_2_). The heart was allowed to beat spontaneously throughout the experiment. To determine left ventricular pressure, a saline-filled latex balloon was inserted into the left ventricle through an incision in the left atrial appendage. The balloon was tied securely into place and filled with saline to give an end-diastolic pressure of approximately 10 − 15 mmHg. The hearts were placed in a water-jacketed heart chamber (Radnotti, Harvard apparatus, USA) maintained at 37 °C and allowed to stabilize for 30 minutes. The intraventricular balloon catheter was connected to a pressure transducer. Left ventricular pressure and heart rate were monitored continuously and recorded on a computer. Digital analysis of the wave was performed and displayed by an electronic polygraph (NEC-San-ei, 2238, Tokyo, Japan). Baseline measurements were recorded at the end of this period. Left ventricular function was assessed by left ventricular developed pressure (LVDP) (peak systolic minus end-diastolic pressure), left ventricular end-diastolic pressure (LVEDP), left ventricular end-systolic pressure (LVESP), maximum rate of pressure rise dp/dt max (as two sensitive indices for contractility), and rate pressure product RPP, the product of heart rate and left ventricular developed pressure (HR × LVDP), which correlates well with the cardiac work. Contractile parameters were recorded at 30 minutes, 60 minutes and 120 minutes.

#### Measurement of serum insulin levels

A rat-specific insulin ELISA kit (Spi-Bio, Bertin Pharma, France) was used to measure serum insulin levels. Spectrophotometric reading was performed between 405 and 414 nm.

#### Measurement of serum fibrinogen level

A rat-specific Bio Med-Fibrinogen kit, Egy-Chem (BioMED Diagnostics, OR, USA) was used to measure serum fibrinogen levels.

#### Detection of transplanted MSCs in the heart

To detect transplanted MSCs in the heart tissue, BM-MSCs and AT-MSCs were labeled with green fluorescent protein (GFP) using EzWay™ Transfection Reagent (Komabiotech, Seoul, South Korea). The manufacturer’s protocol was followed for transfection. GFP-transfected cells were incubated for 24 h prior to testing and then visualized under a fluorescent microscope prior to injection (Figure S2 in Additional file 2). To detect cells in the heart, one month after transplantation myocardial tissue sections were immunostained with anti-GFP antibody and visualized under the microscope. The quantification of transplanted MSCs in the heart was performed by RealTime PCR for human ALU transcripts in the transplanted human MSCs in rat hearts as described previously [25]. Briefly, the hearts were quickly removed and frozen in liquid nitrogen, genomic DNA was isolated from the frozen heart samples, and ALU PCR was performed using TaqMan probes to detect transplanted human MSCs in the rat myocardium.

#### Detection of the CD31^+^ cells

Immunohistochemical studies were carried out according to Wang et al. [26]. Cardiac tissue samples from all the groups were fixed in 10 % formalin for 48 h and the paraffin blocks were prepared. Each sample was cut into 5-μm-thick sections and taken onto poly-lysine-coated slides, air-dried overnight at RT, incubated at 60 °C for 20 minutes, dewaxed in xylene, and rehydrated using different descending concentrations of ethanol. Later, the samples were boiled for 10 − 20 minutes in antigen retrieval solution (0.1 M citric acid, 0.1 M sodium citrate buffer solution, pH 6), cooled at RT for 20 minutes and washed twice in PBS. CD31 staining was carried out by using primary antibody (Abcam) and HRP-labeled secondary antibody. Slides were then incubated with 3,3′-Diaminobenzedine (DAB) chromogen (Lab Vision™) mixture for 5 − 15 minutes at room temperature and then counterstained with Mayer-Haematoxylin for 1 − 3 minutes. Morphometric assessment of the area of CD31^+^ cells was performed using Leica Qwin 500 LTD computer-assisted image analysis software (Cambridge, UK). The measurements were done in 10 high power fields (HPF) in all the experimental groups.

#### Detection of immune cell infiltration and fibrosis

H&E staining was performed to detect immune cell infiltration. Briefly slides were deparaffinized in xylene, rehydrated in different descending concentrations of ethanol and stained in hematoxylin. Next, the slides were counter stained in eosin, dehydrated in increasing concentrations of ethanol and xylene and mounted. The images were captured under the microscope. The percent area of mononuclear infiltrating cells was calculated using the Leica Qwin 500 LTD computer-assisted image analysis software (Cambridge, UK). To detect fibrosis myocardial sections were stained with Masson’s trichrome and the percent area of collagen fibers was calculated using the Leica Qwin 500 LTD computer-assisted image analysis software (Cambridge, UK).

#### Western blotting

Briefly, myocardial tissue protein extracts prepared from control and treated samples in different groups were suspended in PBS containing protease inhibitor cocktail, and 50 μg of protein was loaded onto 10 % TGX FastCast Acrylamide gel (Bio-Rad Laboratories Ltd). Electrophoresis, immunoblotting, and protein detection were done for α-smooth muscle actin (α-SMA) using anti α-SMA (Sigma Adrich) antibody. Bands were visualized with Fluor S-MultiImager MAX system (Bio-Rad Laboratories, Canada) and quantified by image analysis software (Quantity One, Bio-Rad Laboratories, Canada).

#### Statistical analysis

Statistical analysis was done using the software package SPSS. Data are expressed as mean ± SD. Comparisons between groups were done using analysis of variance (ANOVA) and for multiple comparisons the Bonferroni test was used post-test for normally distributed quantitative variables. Quantitative variables that were not normally distributed were compared using non parametric (NPar) tests (Kruskal-Wallis test, Mann-Whitney test). Correlation was assessed to test for linear relationships between quantitative variables. *P* values <0.05 were considered statistically significant.

## Results

### Characterization of MSCs

**Fig. 1:**
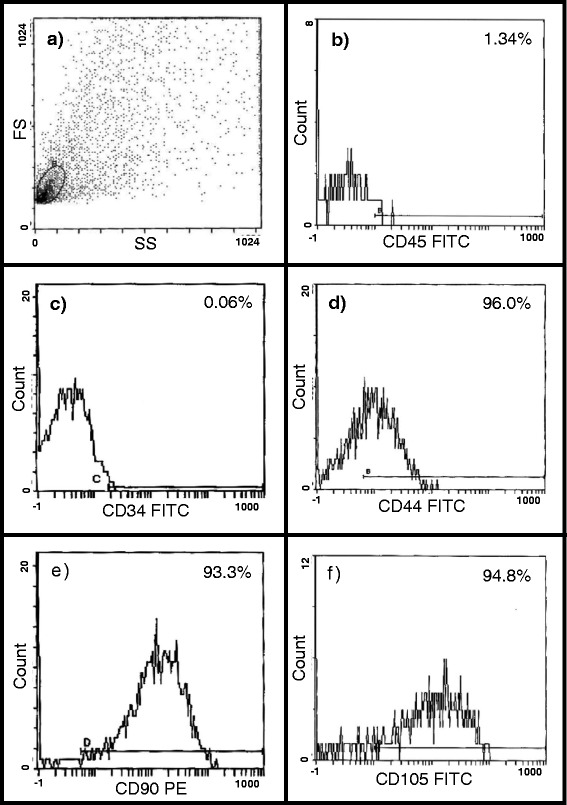
Immunophenotyping of bone marrow-derived mesenchymal stem cells (BM-MSCs) by flow cytometry. **a** Flow gate; **b** CD45; **c** CD34; **d** CD44; **e** CD90 and **f** CD105. All the cells were negative for CD45 and CD34 and >90 % of the cells were positive for CD44, CD90 and CD105. *FS* fractional shortening, *FITC* fluorescein isothiocyanate

To characterize BM-MSCs and AT-MSCs, we performed FACS analysis. Our results demonstrate that >90 % of cells were CD90^+^, CD105^+^ and CD44^+^, while all were CD45^-^ and CD34^-^ (Fig. 1 and Figure S1 in Additional file 3).

### Blood glucose

**Fig. 2:**
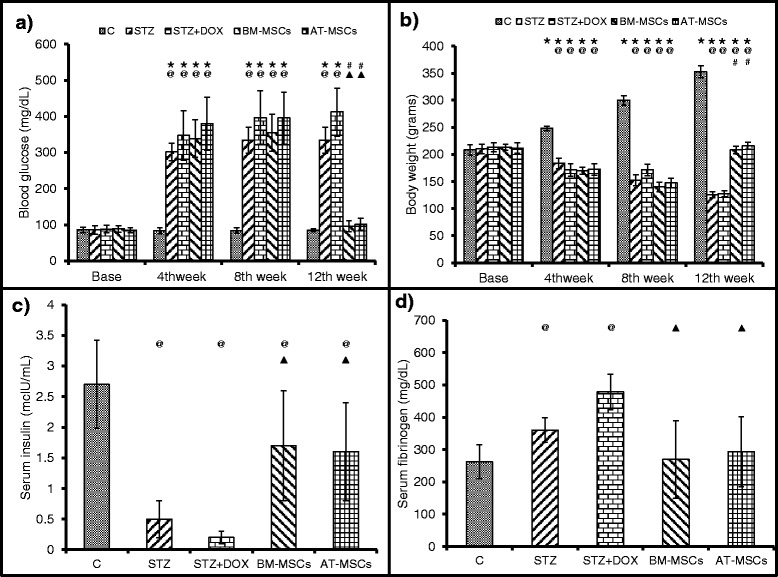
Blood glucose, body weight, serum insulin and fibrinogen levels were measured in different groups. Data are mean ± SD. **a** Blood glucose levels increased after streptozotocin (*STZ*) treatment, and both bone marrow-derived mesenchymal stem cell (*BM-MSC*) and adipose tissue derived mesenchymal stem cell (*AT-MSC*) treatment normalized glucose levels. **b** Body weight increased normally in the control group (*C*) after 4,8 and 12 weeks; in the STZ and the STZ + doxorubicin (*STZ + DOX*) groups body weight decreased at these time points, which was rescued after treatment with BM-MSCs and AT-MSCs. **c** Insulin and **d** fibrinogen levels deviated in the STZ and STZ + DOX groups; treatment with BM-MSCs and AT-MSCs normalized these parameters. *P <0.05 compared to respective baselines, ^@^ *P* <0.05 compared to respective controls at same time points, ^#^ *P* <0.05 compared to respective pretreatment (8 weeks), ^▲^ *P* <0.05 compared to the untreated STZ + DOX group

We measured blood glucose levels in all the groups at baseline and after 4, 8 and 12 weeks after STZ injection to confirm the establishment of diabetes mellitus. There was no difference observed in glucose levels among all the groups at baseline. After 4, 8 and 12 weeks after STZ administration there was a significant increase in blood glucose levels in the STZ and STZ + DOX groups. However, 4 weeks after implantation of BM-MSCs and AT-MSCs (12 weeks after STZ injection), the glucose levels decreased 4-fold in comparison to the STZ + DOX group (Fig. 2a).

### Body weight

We monitored body weight in all the groups at baseline, and 4, 8 and 12 weeks after STZ injection. In the control group, all the animals had a normal growth trend, as there was a significant increase in body weight after 4, 8 and 12 weeks. However in the STZ and STZ + DOX groups body weight decreased after 4, 8 and 12 weeks in comparison to the control group. After 4 weeks of BM-MSC and AT-MSC implantation there was a significant increase in body weight in comparison to the STZ and STZ + DOX groups (Fig. 2b).

### Serum insulin levels

Serum insulin levels were measured in all the groups after 12 weeks of STZ injection. Serum insulin decreased 5-fold in the STZ and STZ + DOX groups in comparison to the control group (Fig. 2c). However, BM-MSC and AT-MSC implantation significantly increased insulin levels in comparison to both the STZ and STZ + DOX group (Fig. 2c).

### Serum fibrinogen levels

Serum fibrinogen levels were measured in all the groups 12 weeks after STZ injection. The levels significantly increased in the STZ and STZ + DOX group in comparison to the control group (Fig. 2d). Both BM-MSC and AT-MSC implantation decreased fibrinogen levels approximately 2-fold (Fig. 2d).

### Arterial blood pressure

**Fig. 3:**
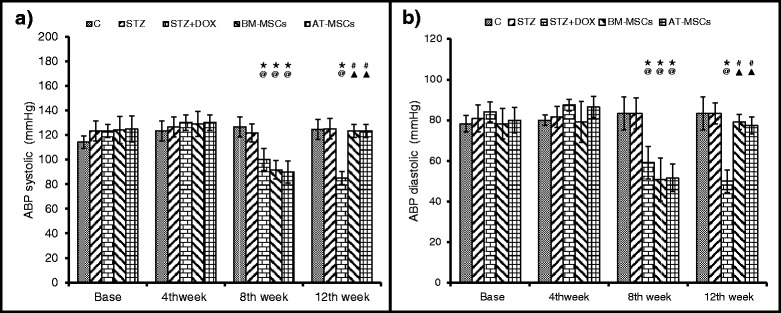
Arterial blood pressure (*ABP*) was measured in different groups. Data are mean ± SD. **a** Systolic ABP and **b** diastolic ABP significantly decreased after 4 weeks of doxorubicin (*DOX*) treatment (8 weeks after streptozotocin (*STZ*) treatment). Both *BM-MSC* and *AT-MSC* treatment rescued ABP levels. *P <0.05 compared to respective baselines, ^@^ *P* <0.05 compared to respective controls (*C*) at time points, ^#^ *P* <0.05 compared to respective pretreatment (8 weeks), ^▲^ *P* <0.05 compared to the untreated STZ + DOX group

ABP was recorded in conscious rats at baseline and 4, 8 and 12 weeks after STZ injection. There was no difference observed among the different groups in systolic or diastolic ABP levels at baseline or after 4 weeks of STZ administration (Fig. 3a, b). However, after 4 weeks of DOX treatment (8 weeks after STZ injection) both systolic and diastolic ABP levels significantly decreased. In both BM-MSCs and AT-MSCs after 4 weeks of implantation (12 weeks after STZ injection) ABP increased 1.5-fold compared to the DOX group (Fig. 3a, b).

### In vivo cardiac function assessment

**Fig. 4:**
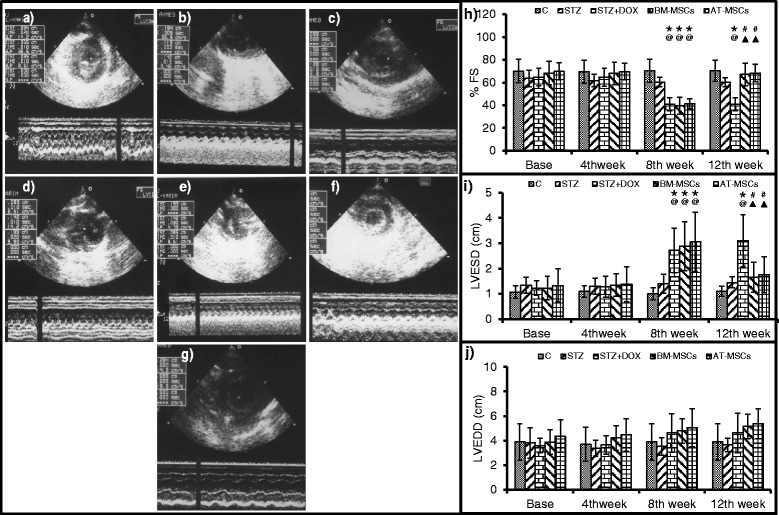
Heart function was assessed in different groups by echocardiography. Data are mean ± SD. **a**-**g** M mode pictures in different treatment groups. **a** Control, **b** streptozotocin (*STZ*), **c** STZ + doxorubicin (*STZ + DOX*, **d** bone marrow-derived mesenchymal stem cells (*BM-MSCs*) before injection, **e** BM-MSCs post-treatment, **f** adipose tissue-derived mesenchymal stem cells (*AT-MSCs*) before injection, **g** AT-MSCs post-treatment, **h** percent fractional shortening (*% FS*), **i** LVESD, and **j** left ventricular end diastolic dimension (*LVEDD*). After 4 weeks of DOX treatment (8 weeks after STZ) heart function deteriorated; both BM-MSC and AT-MSC implantation improved heart function. *P <0.05 compared to respective baselines, ^@^ *P* <0.05 compared to respective controls at time points, ^#^ *P* <0.05 compared to respective pretreatment (8 weeks), ^▲^ *P* <0.05 compared to the untreated STZ + DOX group

To determine the effect of MSC implantation on cardiac function, we performed echocardiography at baseline, and 4, 8 and 12 weeks after STZ injection. There was no difference observed in left ventricular (LV) volumes and percent FS among different groups at baseline and after 4 weeks of STZ treatment (Fig. 4). However, after 4 weeks of DOX treatment (8 weeks after STZ injection) there was a significant increase observed in LV diameter and a substantial decrease in percent FS. In both BM-MSCs and AT-MSCs, 4 weeks of implantation (12 weeks after STZ injection) led to an improvement in heart function as we observed a significant increase in percent FS and a decrease in LV volumes (Fig. 4).

### Isolated heart perfusion

**Fig. 5:**
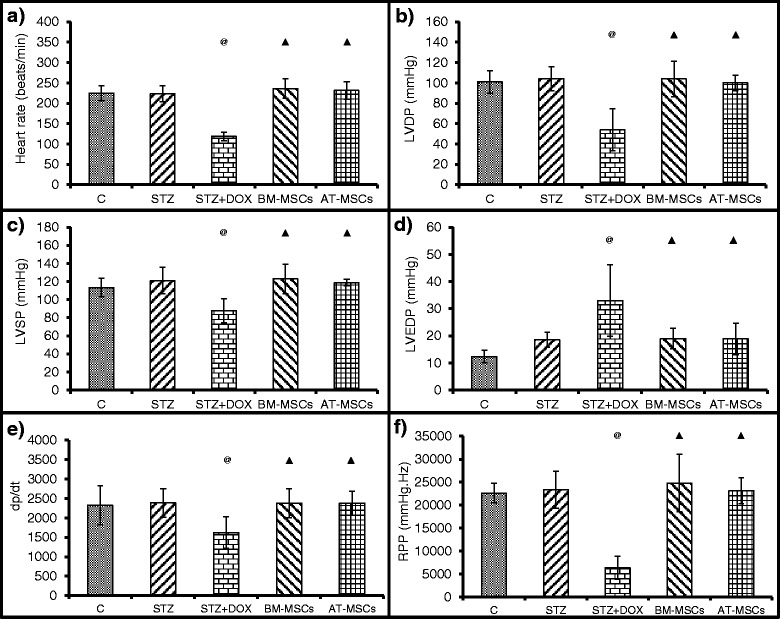
Retrograde Langendorff perfusion was performed on isolated hearts at the end of the study (12 weeks after streptozotocin (*STZ*) injection) to measure **a** heart rate, **b** left ventricular developed pressure (*LVDP*), **c** LVSP, **d** left ventricular end diastolic pressure (*LVEDP*), **e** maximum rate of pressure rise (*dp/dt max*) and **f** rate pressure product (*RPP*) in different groups. Data are mean ± SD. ^@^ *P* <0.05 compared to respective controls at time points, ^▲^ *P* <0.05 compared to the untreated STZ + doxorubicin (*STZ + DOX*) group. *BM-MSCs* bone marrow-derived mesenchymal stem cells, *AT-MSCs* adipose tissue-derived mesenchymal stem cells

Retrograde Langendorff perfusion was performed to measure heart rate, LVESP, LVEDP, LVDP, dp/dt and RPP. There was a 2-fold decrease in heart rate after DOX treatment compared to the control and STZ groups. Both BM-MSC and AT-MSC therapy stabilized the heart rate (Fig. 5a). We observed a significant decrease in LVDP and LVSP, and an increase in LVEDP after DOX administration. Both BM-MSC and AT-MSC treatment improved these parameters (Fig. 5b, c, d). Assessment of cardiac contractility was done by the measurement of dp/dt and RPP levels. DOX administration decreased these parameters, however, both BM-MSCs and AT-MSCs were equally effective in preventing these changes (Fig. 5e, f).

### Detection of stem cells in the heart

**Fig. 6:**
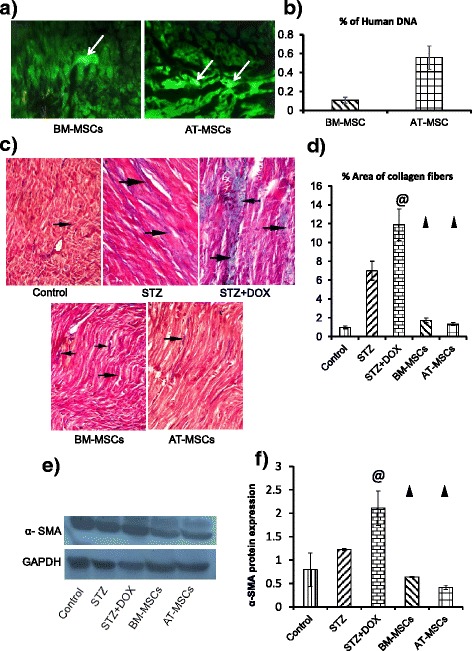
**a** Bone marrow-derived mesenchymal stem cells (*BM-MSCs*) and adipose tissue-derived mesenchymal stem cells (*AT-MSCs*) were labeled with green fluorescent protein (*GFP*) before injection. Implanted cells were detected in the myocardium 4 weeks after intravenous injection through the tail vein (magnification × 10). **b** Real-time PCR was performed to quantify transplanted MSCs in the heart. Genomic DNA was isolated from the heart samples, and ALU PCR was performed using TaqMan probes to detect transplanted human MSCs in the rat myocardium. Histograms show percentage of human DNA found in total DNA extracted from rat heart tissues from the BM-MSC and AT-MSC groups. **c** and **d** Myocardial sections were stained with Masson’s trichrome, (magnification × 200) to detect fibrosis in the heart in the different groups (control, streptozotocin (*STZ*), STZ + doxorubicin (*STZ + DOX*), BM-MSCs and AT-MSCs; **d** histograms show percent area of collagen deposition. *Black arrows* indicate collagen fibers. Quantification of the cells in various groups was performed using the Leica Qwin 500 LTD computer-assisted image analysis system (Cambridge, UK). Measurements were done in 10 high power fields (HPF) in all the experimental groups. **e** and **f** α-smooth muscle actin (*α-SMA*) expression was assessed by western blot in different groups. **f** Histograms show α-SMA levels, values were normalized with glyceraldehyde-3-phosphate dehydrogenase (*GAPDH*). ^@^ *P* <0.05 compared to STZ, ^▲^ *P* <0.05 compared to STZ and STZ + DOX

The ability of injected stem cells to repair the heart depends upon successful delivery of injected cells in the cardiac tissue. We labeled the stem cells with GFP before transplantation (Figure S2 in Additional file 2), our immunohistochemistry results demonstrated that 4 weeks after intravenous injection, implanted BM-MSCs and AT-MSCs were present in the heart (Fig. 6a). Real-time PCR was performed for quantification of MSCs in the heart. We detected 0.1078 % of human DNA in the total DNA extracted from the heart tissues in the BM-MSC group and 0.557 % of human DNA in total DNA extracted from heart tissues in the AT-MSC group (Fig. 6b).

### Assessment of fibrosis and inflammation

To detect fibrosis in the heart, myocardial sections were stained with Masson’s trichrome; control rats had fine collagen fibers between the muscle fibers, diabetic rats (STZ group) had dense collagen fibers between the muscle fibers, and the STZ + DOX group had extensive collagen fibers between disorganized muscle fibers. These results were further complimented by α-SMA expression, which is a very well-established marker of cardiac fibrosis. We observed a significant increase in α-SMA protein levels in the STZ + DOX group in comparison to the control and STZ groups. Both BM-MSC and AD-MSC transplantation decreased the level of fibrosis, as we observed decreased collagen deposition and α-SMA protein levels (Fig. 6c-f).

**Fig. 7:**
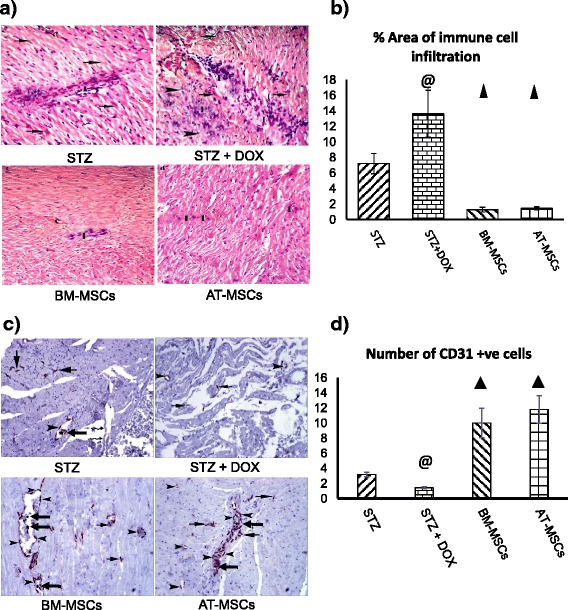
**a** H & E staining was performed to detect infiltration of immune cells in the heart. Photomicrographs of heart sections (×200) from different groups: control, streptozotocin (*STZ*), STZ + doxorubicin (*STZ + DOX*), bone marrow-derived mesenchymal stem cells (*BM-MSCs*) and adipose tissue-derived mesenchymal stem cells (*AT-MSCs*) . *Black arrows* indicate infiltrating immune cells. **b** Histograms show percent area of immune cell infiltration. **c** CD31 expression in the myocardium was examined by immunohistochemistry (magnification × 200) in different groups. *Black arrows* indicate CD31^+^ cells; *wavy arrows* show damaged muscle tissue. **d** Histograms show percentage of CD31^+^ cells. Quantification of the cells in various groups was performed using the Leica Qwin 500 LTD computer-assisted image analysis system (Cambridge, UK). The measurements were done in 10 high power fields in all the experimental groups. ^@^ *P* <0.05 compared to STZ, ^▲^ *P* <0.05 compared to STZ and STZ + DOX

On H&E staining of the heart sections there was high infiltration of immune cells in the STZ and STZ + DOX groups, along with distortion of myocytes (Fig. 7a, b). Both BM-MSC and AT-MSC treatment significantly decreased the infiltration of immune cells (Fig. 7a, b).

### Assessment of angiogenesis

CD31 has been widely relied upon as a marker for angiogenesis. We have tested myocardial tissue in different groups for CD31expression (Fig. 7c). CD31^+^ cells were found in the STZ group (Fig. 7c) and were markedly reduced in the STZ + DOX group (Fig. 7c). Both BM-MSC and AT-MSC treatment increased the number of CD31^+^ cells 5-fold (Fig. 7c, d).

## Discussion

In the present study, we report for the first time that mesenchymal stem cell therapy prevents DOX-induced deterioration of cardiac function in diabetic rats. In various pre-clinical and clinical studies, AT-MSCs and BM-MSCs have comparable outcomes [27, 28]. Therefore we investigated both AT-MSCs and BM-MSCs to assess their respective efficacy. Both were equally effective in mitigating DOX-induced alterations in cardiac function in diabetic rats.

Previously, several studies have demonstrated that MSC transplantation in diabetic rats downregulates hyperglycemia and normalizes body weight [29, 30]. Similarly, we found that AT-MSC and BM-MSC transplantation decreased glucose levels and increased body weight in STZ-induced diabetes in rats. However, it was unclear until now whether MSC therapy can decrease blood glucose levels in DOX-treated diabetic rats. Our experiments reported a significant decrease in blood glucose levels in DOX-treated animals. It has been suggested that insulin and fibrinogen play a key role in glycemic control in the body [31]. To determine if this may be the cause of MSC therapy-mediated decrease in blood glucose levels, we measured serum insulin and fibrinogen levels. It was found that serum insulin levels decreased and fibrinogen levels increased in STZ-treated and DOX-treated animals. AT-MSC and BM-MSC injections both restored the levels of insulin and fibrinogen to those found in the control group.

As diabetic patients are more susceptible to cancer than normal people, DOX therapy becomes a necessary evil, threatening to cause a severe degree of damage to the already ailing heart of diabetic patients. This was amply substantiated in the current study. We found significant deterioration of cardiac function in the DOX-treated STZ group compared to the control and STZ-treated animals. We investigated a number of parameters to assess cardiac damage after treatment with doxorubicin. Heart rate has been ascertained as an independent risk factor in cardiovascular disease [32]. We found a significant decrease in the heart rate in the DOX-treated group. Both BM-MSC and AT-MSC transplantation prevented this decrease. Our experiments further demonstrated that stem cell transplantation prevents DOX-induced decrease in percent FS and improved LV dimensions.

The RPP represents the myocardial workload, providing a direct indication of the energy demand and energy consumption of the heart, which can digress in cardiovascular risk [33]. We observed A significant decrease in RPP levels after 4 weeks of DOX treatment. However, stem cell implantation increased RPP levels. For further assessment of the effects of BM-MSCs and AT-MSCs in modulating cardiac function, hearts from all the experimental groups were isolated and perfused using Langendorff apparatus and dp/dt was evaluated to assess the cardiac contractility of the isolated perfused hearts. dp/dt has been established as a predictor of event-free survival in patients with heart failure [34]. We found both AT-MSC and BM-MSC therapy prevented DOX-induced decrease in dp/dt levels. In various animal models the transplantation of mesenchymal stem cells to the damaged myocardium has been found to improve heart function and prevent congestive heart failure [35–38].

DOX is known to cause disruptions of cardiac function through different mechanisms. Several studies have reported the impairment of angiogenesis, increase in inflammatory cell infiltration and fibrosis following DOX therapy [39, 40]. The suggested mechanisms of MSC-mediated cardiac repair include modulation of inflammation [41] and promotion of angiogenesis [42]. Therefore, in this study, to find the mechanisms of stem cell therapy-mediated protection against DOX-induced damage, we assessed the level of angiogenesis, immune cell infiltration and fibrosis following MSC therapy. MSCs have been regularly reported to release angiogenic factors such as SDF-1, VEGF, PIGF, FGF-2, angiopoeitin-1, PDGF, MCP-1, plasminogen activator and MMP-9 [12, 13, 43, 44]. Our study further substantiates the notion of MSC-induced angiogenesis in damaged myocardium, helping in improvement of cardiac function, thus strongly corroborating previous studies wherein MSCs were found to stimulate angiogenesis in animal models of myocardial infarction, which eventually resulted in repair of ischemic heart tissue [45, 46]. MSCs also secrete various immunosuppressive soluble factors such as prostaglandin E2 (PGE2), IL-10 and indolamine dioxygenase (IDO) [47]. These factors modulate inflammatory responses in the infarcted heart and promote the repair process. In fibrosis, there is excessive fibroblast accumulation, extracellular matrix (ECM) deposition and scar formation, leading to increased stiffness of the heart tissue, culminating in progressive cardiac failure [48]. Reducing cardiac fibrosis is critical in improving the condition of the affected heart [49]. Our findings are in agreement with the previously published reports and show that there was a marked reduction in fibrosis in the BM-MSCs and AT-MSC groups compared to the STZ + DOX group. Some of the initial studies have also demonstrated that mouse bone marrow MSCs trans-differentiated into cardiomyocytes in the heart [50]. However, subsequent studies have revealed that cardiac differentiation of MSCs is limited to the expression of cardiac-specific markers, without the generation of functional cardiomyocytes [51, 52].

Based on the outcome of these pre-clinical studies, a wide variety of MSC-based clinical trials have reported improvement in function in patients with cardiovascular and metabolic disease [53–56]. However, before making definitive conclusions and applying MSCs for widespread clinical use, there is a need for more studies to understand the in-depth mechanisms and the duration of improvement in patients treated with stem cell therapy.

## Conclusions

In conclusion, the present study not only confirms the antidiabetic potential of MSCs, but we also show for the first time that both AT-MSCs and BM-MSCs are equally effective in restoring heart function in DOX-treated diabetic rats. Furthermore, these findings should act as a stimulus for further research on the benefits of mesenchymal stem cell therapy for diabetic patients suffering from cancer.

## Additional files

Additional file 1: Figure S3. Schematic diagram of the timeline of all the groups. (PDF 11 kb) Additional file 2: Figure S2. Figure S2 Representative image of a stem cell expressing the transfected green fluorescent protein prior to injection (magnification × 20). (PDF 13 kb) Additional file 3: Figure S1. Immunophenotyping of adipose tissue-derived mesenchymal stem cells by flow cytometry. a Flow Gate; b CD45; c CD34; d CD44; e CD90 and f CD105. All the cells were negative for CD45 and CD34; >90 % of the cells were positive for CD44, CD90 and CD105. (PDF 164 kb)

## Abbreviations

α-MEM: alpha minimum essential media
α-SMA: α-smooth muscle actin
ABP: arterial blood pressure
AD: adipose tissue
ANOVA: analysis of variance
ASCs: adipose stem cells
AT-MSCs: adipose tissue-derived mesenchymal stem cells
bFGF: basic fibroblast growth factor
BM-MSCs: bone marrow-derived mesenchymal stem cells
DAB: 3,3′-Diaminobenzedine
DMEM: Dulbecco’s modified Eagle’s medium
DOX: doxorubicin
dp/dt: rate of pressure rise
ELISA: enzyme-linked immunosorbent assay
FACS: fluorescence-activated cell sorting
FBS: fetal bovine serum
FGF-2: fibroblast growth factor-2
FITC: fluorescein isothiocyanate
FS: fractional shortening
GFP: green fluorescent protein
H&E: hematoxylin and eosin
HR: heart rate
HRP: horseradish peroxidase
IGF: Insulin-Like Growth Factor
i.p.: intraperitoneal
i.v.: intravenous
IDO: indolamine dioxygenase
IL-10: interleukin 10
ISCT: International Society for Cellular Therapy
KH: Kreb-Henseliet
LV: left ventricle/ventricular
LVDP: left ventricular developed pressure
LVEDD: left ventricular end diastolic dimension
LVEDP: left ventricular end diastolic pressure
LVESD: Left Ventricular End Systolic Diameter
LVESP: left ventricular end systolic pressure
LVSD: left ventricular end systolic dimension
LVSP: Left Ventricular Systolic Pressure
MCP-1: monocyte chemotactic protein-1
MMP-9: matrix metalloproteinase-9
MNCs: mononuclear cells
MSCs: mesenchymal stem cells
NEAA: nonessential amino acids
NPar: non parametric tests
PBS: phosphate-buffered saline
PCR: polymerase chain reaction
PDGF: platelet-derived growth factor
PGE2: prostaglandin E2
PIGF: placenta derived growth factor
RPP: rate pressure product
RT: room temperature
SCF: Stem Cell Factor
SDF-1: stromal cell derived factor-1
STZ: streptozotocin
SVF: stromal vascular fraction
VEGF: vascular endothelial growth factor
