# Supplementary figures and images for: Comparison of adipose tissue- and bone marrow- derived mesenchymal stem cells for alleviating doxorubicin-induced cardiac dysfunction in diabetic rats
Source: Stem Cell Res Ther. 2015 Aug 22;6(1):148. doi: 10.1186/s13287-015-0142-x (PMC4546321; doi:10.1186/s13287-015-0142-x)

# Weeks

Group 1  
Control

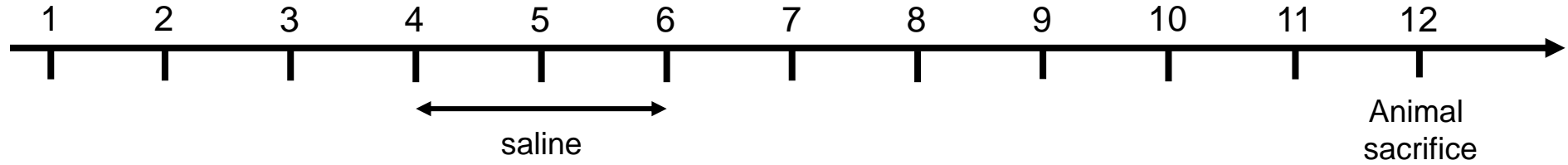

Group 2  
STZ

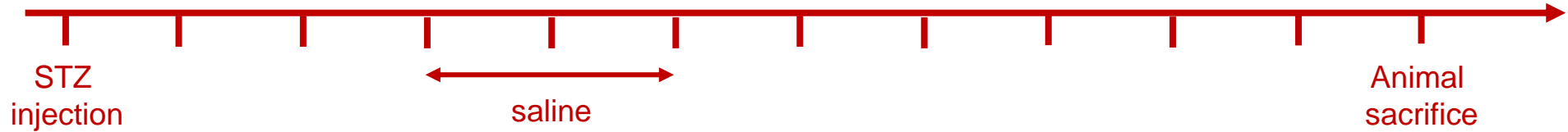

Group 3  
STZ+DOX

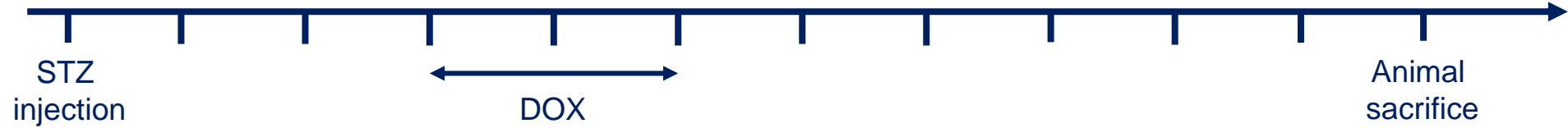

Group 4  
BM-MSCs

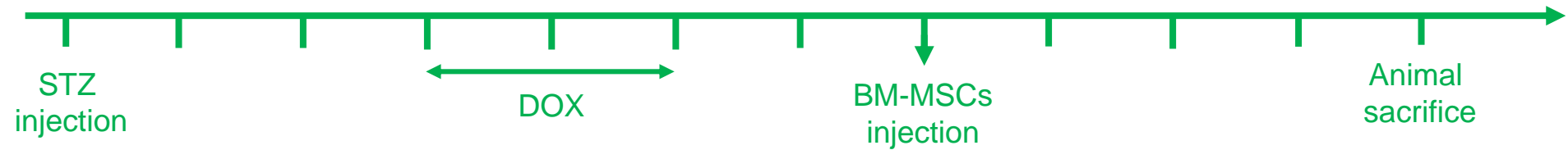

Group 5  
AT-MSCs

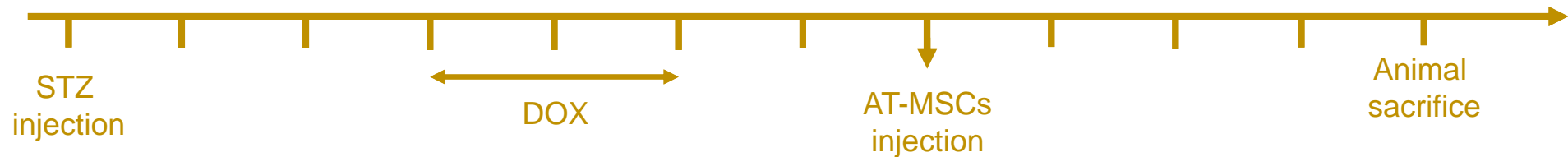

Supplement: Additional file 1: Figure S3. — Schematic diagram of the timeline of all the groups. (PDF 11 kb) [file 13287_2015_142_MOESM1_ESM.pdf]

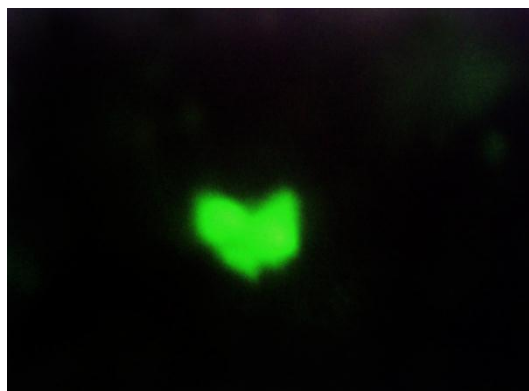

Supplementary Fig. 2 Ammar *et al*

Supplement: Additional file 2: Figure S2. — Figure S2 Representative image of a stem cell expressing the transfected green fluorescent protein prior to injection (magnification × 20). (PDF 13 kb) [file 13287_2015_142_MOESM2_ESM.pdf]

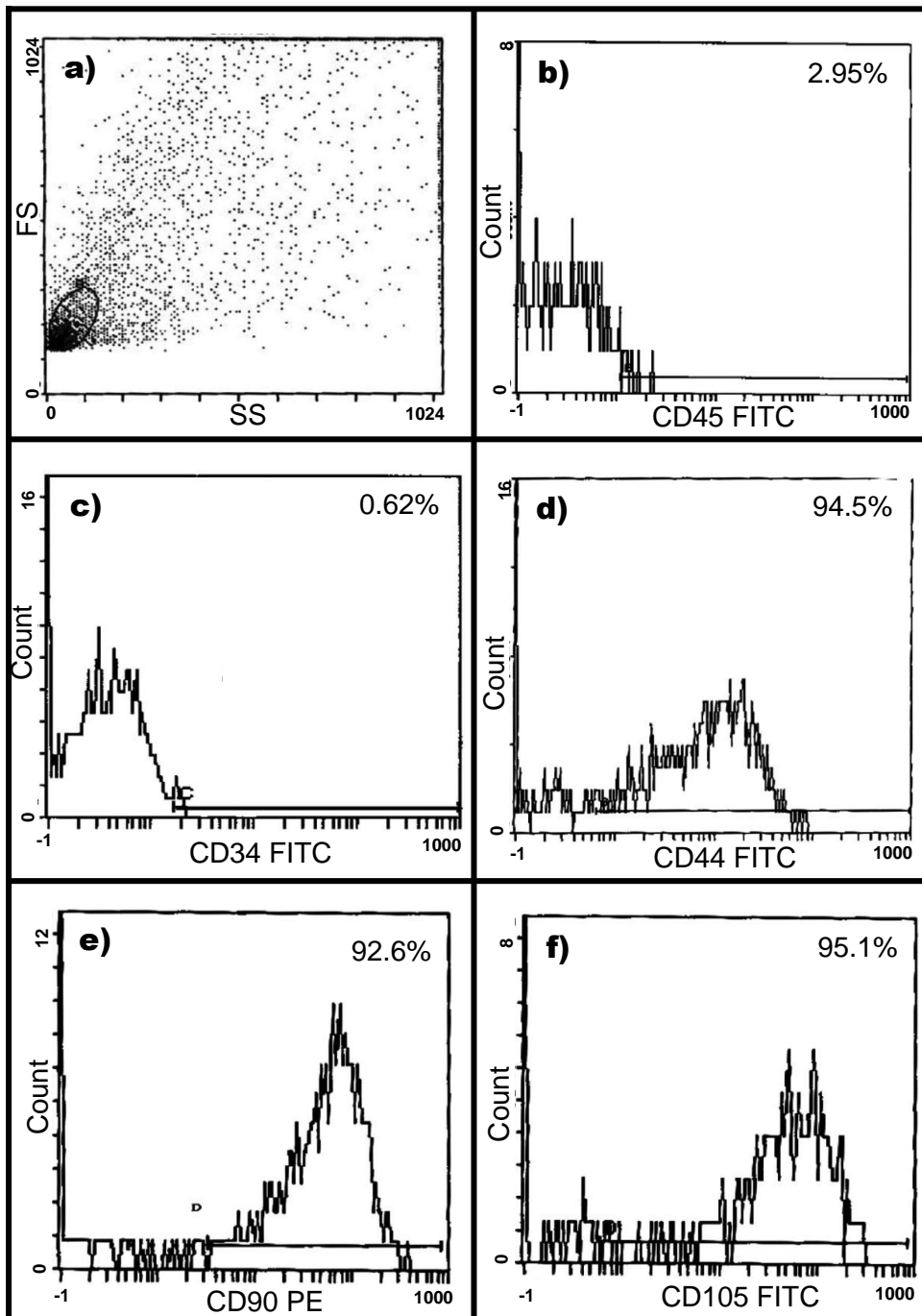

Supplementary Fig. 1 Ammar *et al*

Supplement: Additional file 3: Figure S1. — Immunophenotyping of adipose tissue-derived mesenchymal stem cells by flow cytometry. a Flow Gate; b CD45; c CD34; d CD44; e CD90 and f CD105. All the cells were negative for CD45 and CD34; >90 % of the cells were positive for CD44, CD90 and CD105. (PDF 164 kb) [file 13287_2015_142_MOESM3_ESM.pdf]
